# The Revised Child-to-Parent Aggressions Questionnaire: an Examination During the Covid-19 Pandemic

**DOI:** 10.1007/s10896-022-00465-8

**Published:** 2022-11-12

**Authors:** Esther Calvete, Aitor Jiménez-Granado, Izaskun Orue

**Affiliations:** Department of Psychology, Faculty of Health Sciences, University of Deusto, Avenida de las Universidades, 24, 48007 Bilbao, Spain

**Keywords:** Child-to-parent violence, Adolescents, COVID-19 pandemic, Reasons for aggressions

## Abstract

**Purpose:**

Child-to-parent violence (CPV) is an important type of family violence that has been relatively understudied. This study examined the main psychometric properties of the revised Child-to-Parent Aggression Questionnaire (CPAQ-R), which examines both violent behaviors against parents and reasons for these behaviors. The aims included identifying the dimensions of CPV and examining the magnitude of CPV during the COVID-19 pandemic.

**Methods:**

A sample of 1,244 adolescents (aged from 12 to 17) from several schools in the Basque Country completed the CPAQ-R. Several confirmatory factor analyses were conducted, including exploratory, confirmatory, exploratory structural equation modeling (ESEM), and bifactor analyses.

**Results:**

The data supported a bifactor ESEM model in which a general factor of violence against parents explained aggressions against both mothers and fathers. In addition, three reasons for the violence emerged: instrumental, reactive, and defensive reasons. Rates of CPV during the COVID-19 pandemic were high, with 16.5% of adolescents reporting reiterative aggressions against their parents. There were no differences between aggressions against mothers and fathers.

**Conclusions:**

The CPAQ-R is an adequate questionnaire for assessing CPV in adolescents. The confinement and restrictions placed on families during the COVID-19 pandemic may explain the high prevalence of CPV and shed light on possible differences related to the sex of the parents.

Child-to-parent violence (CPV) by adolescents is a relevant type of family violence. Recently, based on a committee of experts, the Spanish Society for the Study of Child-to-Parent Violence defined CPV as repeated behaviors of physical, psychological (verbal or nonverbal), or economic violence directed at parents or those who take their place (Pereira et al., 2017). CPV has important clinical and social repercussions. It causes enormous suffering for the victims, who may experience psychological problems and social stigma (Williams et al., 2017), and is also often an indicator of the presence of other family problems, such as exposure to family violence (Calvete et al., 2015c), and individual psychological problems in children, such as drug use and depression (Calvete et al., 2020; del Hoyo et al., 2020; see Simmons et al., 2018 for a review).

## Prevalence of CPV in Adolescents

According to the Report of the State Attorney General’s Office, 4,699 cases were opened against young people for CPV in Spain in 2020 (Fundación, 2021). However, these data may represent only the tip of the iceberg. Many parents do not report or ask for help for this problem due to social stigma, as they fear that the occurrence of CPV in their family could be interpreted as a result of a failure in parental education and setting limits for their children (Calvete et al., 2017; Ibabe, 2019). This contributes to CPV being perhaps the most underreported type of domestic violence (Brule & Eckstein, 2016). Therefore, it is believed that significant proportion of CPV cases remain hidden (Condry & Miles, 2014).

Data from community samples help to shed light on hidden CPV in society, which generally does not reach the courts or specialized centers. However, the data obtained are somewhat incongruent. In their review, Simmons et al. (2018) found prevalence rates between 5 and 21% for physical CPV and 33 to 93% for psychological CPV in community samples. In another review, it was recommended that the data obtained in many of the studies should be considered with caution for two reasons (Calvete, 2019). The first is because they are based on the criterion that at least one aggressive behavior took place in the last year. This criterion is clearly insufficient according to the definition of CPV, which highlights the repeated nature of the aggressions (Pereira et al., 2017). The second reason is that some of the behaviors included in the questionnaires are relatively common in adolescence (e.g., yelling at the mother or father in a moment of anger or doing something in order to annoy them). Some of these behaviors, especially when they occur in isolation, could be an expression of rebelliousness and a desire to achieve a certain autonomy from adults, which are characteristic of adolescence (Calvete, 2019). When stricter criteria are incorporated and in accordance with the definition of CPV, the percentages are much lower. In one study applying stricter criteria, the rate of psychological violence decreased from 92.7 to 14.2% and that of physical violence from 10.7 to 3.2% (Calvete et al., 2013a). Notably, these rates are considerably higher in clinical and judicial samples (Del Hoyo-Bilbao et al., 2018; Ibabe et al., 2014).

The sex of the persons involved in CPV has received considerable attention. As for the sex of the children, numerous studies with community samples suggest that there are no differences between boys and girls (Ibabe & Bentler, 2016; Pagani et al., 2009), and there may even be a higher frequency of psychological aggression by girls than by boys (Margolin & Baucom, 2014). In samples of offenders, more cases are found in boys than in girls (Armstrong et al., 2018; Ibabe & Jauregizar, 2010; Simmons et al., 2018).

Although there are exceptions, in the majority of studies mothers are overwhelmingly reported to be the primary targets of CPV in community and clinical samples (Simmons et al., 2018). Two main reasons for these differences have been suggested. First, they could involve social learning of the aggressions perpetrated against mothers by their partners, of which the children are often witnesses (Ibabe et al., 2020). Second, there is a greater presence of mothers in child rearing, so they are more often placed in the context of the conflicts and discussions that precede aggressive acts (Ulman & Straus, 2003).

### Child-To-Parent Violence During the Pandemic

The scenario brought about by the pandemic has led to a greater presence of fathers in child rearing. Home confinement and the implementation of telework in many homes means that fathers, mothers, and children share much more time at home. This change has resulted in an increase in family violence (Usher et al., 2021). For instance, one study found that family violence victimization increased during the COVID-19 pandemic (Drotning et al., 2022). However, this study did not examine who the perpetrator of the violence was, as the data only referred to the occurrence of various types of violence perpetrated by a member of the household against another member of the household. Another study found an increase in intimate partner violence during the pandemic (Arenas-Arroyo et al., 2021). Regarding CPV, in a study of young adults (18–25 years) in Spain (Cano-Lozano et al., 2021), the participants reported having engaged in at least one aggressive behavior toward their parents in the last month (65.2% toward the mother and 59.4% toward the father). However, this study focused on young adults, and previous research indicates that the peak of both child-to-mother violence (CMV) and child-to-father violence (CFV) occurs around the age of 15 (Calvete et al., 2020). The only available study regarding CPV perpetrated by adolescents during the COVID-19 pandemic was conducted by Condry et al. (2020), who evaluated a small sample of parents of children (10–19 years) who were violent or abusive in the family. Specifically, they considered the parents’ experiences of victimization during the first months of the pandemic and found that 70% of the parents reported an increase in the number of CPV episodes during the lockdown. In the same study, the researchers consulted practitioners, 69% of whom said they had seen an increase in referrals for families experiencing CPV (Condry et al., 2020). Although these data suggest that CPV may have worsened during the pandemic, to the best of our knowledge no study has evaluated it based on adolescents’ reports.

### Assessment of CPV

Assessment of CPV is essential to determine the magnitude of the problem and to establish appropriate interventions (Calvete, 2019; O’Hara et al., 2017). Many of the pioneering studies on CPV employed the CTS-CP scales (Conflict Tactics Scale Children-Parent; Straus et al., 1996) to assess abusive child-to-parent behaviors. Since then, researchers have used their own adaptations of the CTS, adjusting the number of items and response format based on their research objectives (e.g., Straus & Fauchier 2008; Ullman & Straus, 2003). One such version was developed by Straus and Fauchier (2008) in the context of the International Parenting Study. This version consisted of a three-item verbal aggression subscale (name-calling, yelling, and threatening to hit parents) and a three-item physical aggression subscale (slapping, hitting with an object that can cause harm, and kicking parents). This version, often with modifications, has been used in numerous studies (for reviews, see Calvete 2019; Ibabe, 2020).

Subsequently, several questionnaires have been developed for the assessment of CPV (for reviews, see Arias-Rivera et al., 2020; Ibabe, 2020). Specifically, the Child-to-Parent Aggression Questionnaire (CPAQ; Calvete et al., 2013a) is considered a promising instrument that provides valuable information for assessment and intervention in situations of CPV, although more information on its methodological quality is still needed (Arias-Rivera et al., 2020; Ibabe, 2020). In its initial version, the CPAQ included two sections: behaviors and reasons. The behaviors section consisted of 10 parallel items (for aggressions against the mother and against the father). The behavioral items included both physical aggression (three items; e.g., “You kicked or punched your mother/father” and “You hit your mother/father with something that could hurt her/him”) and psychological aggressions (seven items; e.g., “you yelled at your mother/father when you were angry” and “you insulted or said bad words to your mother/father”). The reasons section included 10 items that were answered four times (for physical and psychological aggression and for mother and father). The items included instrumental (four items; e.g., “to get permission for something”), affective (four items; e.g., “because I was very angry”), and defensive (two items; e.g., “to defend myself”) reasons. The factor structure of the CPAQ has been confirmed in community (Calvete et al., 2013a) and clinical (del Hoyo et al., 2018) samples. CPAQ scores are positively correlated with other measures of general aggression (Calvete et al., 2015b) as well as proactive and reactive aggression (Calvete et al., 2013b). The psychological and physical aggression scales have shown adequate internal consistency indices (e.g., between 0.73 and 0.87; Calvete et al., 2013a; Del Hoyo et al., 2018). Similarly, alpha coefficients for the reasons scales have been reported between 0.87 and 0.91 (Calvete & Orue, 2016).

Although the CPAQ has shown good psychometric properties in terms of confirmatory structure and reliability, there are a number of factors that suggest that a revision and updating of its contents is necessary. First, the CPAQ was inspired by the CTS scales developed by Straus et al. (1996). Since then, numerous social and economic changes have occurred. It has been proposed that these changes have influenced CPV behaviors. For example, adolescents have been associated with greater levels of consumerism (Passini, 2013) and materialism (Flurry et al., 2021), and it has been found that episodes of CPV often occur in contexts in which children ask their parents for money and the parents refuse (Calvete et al., 2015d). Second, adolescence is a developmental stage characterized by the desire for autonomy from adults and rebelliousness when young people perceive that parents are attempting to impose behavioral norms on them (Yeager et al., 2018). Some items included in the CPAQ can be considered as manifestations of this rebelliousness characteristic of adolescence, which explains the high percentage of adolescents who indicate that they have engaged in these behaviors. For example, the behavior of yelling at a parent when angry has been found in more than 92% of adolescents in community samples (Calvete & Orue, 2016). Finally, the reasons section can be considered particularly long, given that its 10 items are answered up to four times depending on the responses to the behaviors section. Therefore, this section would also benefit from being shortened.

### The Current Study

As explained above, there are important reasons to update the CPAQ. In this study we revised the CPAQ and developed a new version (CPAQ-R). This involved eliminating items that were questionable as indicators of CPV, adding new items more characteristic of current CPV, and reducing the reasons section in order to make the questionnaire less time-consuming to answer. In making these changes, we relied on materials obtained using a qualitative methodology with samples of adolescents who had perpetrated CPV and their parents (Calvete et al., 2015d) and we followed the recommendations of a committee of expert CPV clinicians and researchers (Pereira et al., 2017). The objectives of the study were to evaluate the psychometric properties of the new version and to provide data on the prevalence of the problem in Spanish adolescents during the COVID-19 pandemic. Given the situation created by the pandemic, the data could contribute to a better understanding of CPV.

## Method

### Participants

The sample was made up of 1,244 adolescents (49.7% boys, 49.4% girls, and 0.9% non-binary; aged from 12 to 17; *M*_age_ = 14.01, *SD* = 1.20) from six high schools in Bizkaia (Basque Country, Spain). Regarding nationality, 93.8% of the participants were Spanish, 6% were foreigners mainly from South American countries, and 0.2% did not report their nationality. With regard to family structure, 80.3% of the participants lived with both parents (plus 8.2% who lived separately with both parents in any regime of shared custody), 8.4% lived with only the mother or the mother and a new partner of the mother, 1.5% lived with the father or the father and a new partner of the father, 0.7% lived in another context (with other relatives or in an educational context), and 0.9% did not report.

According to the criteria of the National Institute of Statistics of Spain, the socio-economic status of the participants was as follows: restaurant and security service workers and vendors (20.55%), scientific and intellectual professionals (19.18%), artisans and skilled workers in the manufacturing and construction industries (17.25%), elementary occupations (10.81%), accounting and administrative employees (9.69%), housekeepers (7.33%), technicians and support professionals (5.74%), machinery operators (3.81%), directors and managers (2.91%), unemployed (2.02%), and skilled workers in the agricultural, livestock, forestry, and fishing sectors (0.71%).

### The Child-to-Parent Aggressions Questionnaire-Revised

The original CPAQ consists of two sections. The first section assesses violent behaviors while the second section assesses reasons for these behaviors. The behaviors section includes 20 parallel items, 10 relating to the father and 10 relating to the mother. Adolescents have to indicate how often they had engaged in each of the behaviors against their fathers or mothers or against the caregivers who adopted the role of their parents (stepmother, stepfather, grandparents, etc.) in the past year using the following four-point scale: 0 (*never*); 1 (*it has happened once or twice*); 2 (*it has happened between three and five times*); and 3 (*it has happened six or more times*). The second section includes 10 parallel items (for behaviors against mothers and fathers) and is only for adolescents who indicate having engaged in at least one violent behavior. The response options range from 0 (*never*) to 3 (*almost always*).

We made the following changes in the first section: the items describing the behaviors of yelling when angry, taking money without permission, using blackmail to get what one wants, and disobedience were eliminated. The reason for eliminating them is that they may represent rebellious and/or rule-breaking behaviors that are relatively typical of adolescence, which do not necessarily involve violence. In addition, we added four items describing behaviors of ridiculing, saying something humiliating or disqualifying, breaking an object in the home during an argument with the parent, and making the parent feel frightened during an argument. As mentioned, these items were included from material obtained in qualitative studies with adolescents and parents involved in CPV (Calvete et al., 2015d) and professionals working with them (Pereira et al., 2017). Thus, the first section consisted of 18 items (nine focused on the mother and nine focused on the father). Regarding the contents of the items, four items describe physical aggression (e.g., “You’ve pushed or hit him/her in a fight”), three items describe verbal aggression (e.g., “You’ve said something humiliating or disqualifying to him/her”), and two items describe threatening or frightening behaviors (e.g., “You’ve made him/her feel scared in an argument”).

The reasons section also underwent some changes. In order to shorten the application time in the new version the items are answered only once in reference to violence against parents in general. In addition, in the new version some items are grouped together. Thus, the items “to get permission for something,” “to be able to use the computer or cell phone,” and “because of the time to get home” are grouped into the same item: “to get permission for something” (e.g., time to get home, to be able to use the cell phone). In addition, we eliminated items that were not frequently found in the stories of adolescents interviewed in a qualitative study (Calvete et al., 2015d): “Because I felt misunderstood” and “Because my character is like that.” Finally, we added the item “Because I lost control.” Thus, the reasons include seven double items, which are answered both in reference to violent behaviors against the mother and against the father.

### Procedure

The project was approved by the Research Ethics Committee of (masked for review). After receiving approval, 18 high schools from Bizkaia (Spain) were invited to collaborate in the study. Of them, six high schools agreed to participate. Informed consent was required from parental figures (or legal guardians) and the adolescents themselves. Between 2 and 3% of the parents declined their sons’ or daughters’ participation. Measures were collected between October 2020 and June 2021. The questionnaires were answered online through Qualtrics®, and the researchers were present in the classrooms with the adolescents in five of the high schools. Adolescents completed the CPAQ-R in the context of a bigger study, and they needed 25–50 min to answer the whole questionnaire. Due to COVID-19, one of the high schools preferred to limit face-to-face meetings and to answer the questionnaires fully online, with researchers on the video call to help if needed.

### Data Analysis

To examine the factor structure of the behaviors part of the CPAQ we used factor analysis (EFA), confirmatory factor analysis (CFA), and exploratory structural equation modeling (ESEM) with target rotation. The data set (*N* = 1,244) was randomly split into two subsamples. One of these was used for the EFA (*N* = 614), and the other was used for the CFA and ESEM analyses (N = 630). Whereas EFA serves to explore the factor structure of a questionnaire when there is no a priori hypothesis, CFA and ESEM with target rotation serve to test a previously hypothesized structure (Morin et al., 2016). There were no differences between the subsamples in terms of the age and sex of the participants.

Data were treated as categorical, and all factor analyses were conducted with Mplus 8.8 (Muthén & Muthén, 2021) using a robust variance-adjusted weighted least squares estimator (WLSMV). EFAs were performed by GEOMIN rotation for the behavioral items toward the father and toward the mother separately and for all together in subsample 1. Subsequently, in subsample 2, the resulting models in subsample 1 were tested by CFA and ESEM. In the nonhierarchical CFA models, each item was specified to load only on the factor it was designed to measure, with correlations between factors freely estimated. The ESEM models were specified with similar factor loading patterns as in their CFA analogs. However, instead of setting the cross-loadings to zero, we used target rotation, where all cross-loadings are freely estimated but “targeted” to be as close to zero as possible (Asparouhov & Muthen, 2009).

**Fig. 1:**
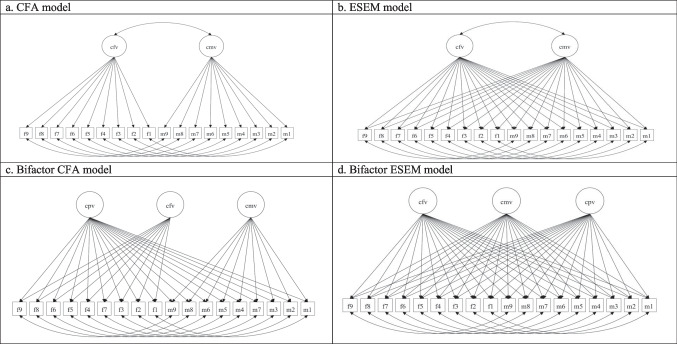
Estimated models. **a **CFA model. **b **ESEM model. **c **Bifactor CFA model. **d **Bifactor ESEM model. Note. CPV = Child-to-Parent violence; CFV = Child-to-Father violence; CMV = Child-to-Mother violence

In addition, we estimated bifactor models. In bifactor CFA models, all items were allowed to load on both a general factor and the corresponding specific factor. In the bifactor model, the covariances between the general and specific factors were set to zero. Bifactor models represent a top-down paradigm in which general factors explain a higher variance of observed variables and specific factors explain residual variance that is not explained by general factors (Markon, 2019). Bifactor models are preferable when they improve fit indices in comparison with lower-order correlated factor models and both the general and specific factors are well defined. Support for a bifactor model allows reporting scores for both a single scale and sub-scores for specific subscales of the questionnaire. Figure 1 displays the estimated models.

Measurement errors for parallel items were allowed to covary in all the models. Thus, we included the covariation between residuals of items assessing the same behavior targeted toward the mother and the father. The percentage of missing values at the item level was insignificant (0.64%). According to some experts, when the percentage of missing values is low—5% or less—almost any procedure for handling missing values yields similar results (Tabachnick et al., 2007). In this case, as we used the WLSMV method, the pairwise procedure in Mplus 8.8 was used to handle the missingness.

The goodness of model fit was evaluated using the comparative fit index (CFI), the Tucker–Lewis index (TLI), the standardized root-mean-square residual (SRMR), and the root-mean-square error of approximation (RMSEA). CFI and TLI values ​​of 0.95 or higher indicate an excellent fit. SRMR and RMSEA values ​​lower than 0.08 indicate a good fit for longitudinal research (Little, 2013). We considered changes in RMSEA greater than 0.015 and changes in CFI and TLI greater than 0.01 as significant in comparing the measurement models (Chen, 2007; Cheung & Rensvold, 2002).

In order to evaluate reliability, for nonhierarchical models, ordinal alpha was estimated. For bifactor models, Omega (ω) coefficients were estimated using the Omega program (Watkins, 2013). Omega total (ω) is an estimation of the proportion of total variance attributable to all sources of common variance. Omega of a subscale (ωs) is the proportion of the subscale score variance that can be attributed to all common factors (i.e., the general factor plus the specific factors). Omega hierarchical (ωH) is the proportion of total score variance that can be attributed to the general factor after accounting for all specific factors. Although there is no consensus regarding cut-offs for ω, it has been proposed that a minimum would be greater than 0.50, and values closer to 0.75 would be preferable (Reise et al., 2013). The Omega Hierarchical Subscale (ωHS) is the proportion of the subscale score variance that can be attributed to the specific factor after accounting for the general factor. Values lower than 0.50 would indicate that the majority of the variance in the subscale’s score is due to the general factor and that insignificant unique variance is due to the specific factor (Hammer & Toland, 2016). In addition, H, explained common variance (ECV), and the percentage of uncontaminated correlations (PUC) were estimated. High H values (> 0.70) indicate that replicability across samples is likely (Rodriguez et al., 2016). ECV is an indicator of the proportion of variance accounted for by all factors that is explained by the general factor, whereas PUC is an indicator of the proportion of correlations that are accounted for by the general factor. When both PUC and ECV are high (> 0.70), a strong general factor is supported, in which case a unidimensional model may be a more parsimonious solution (Rodriguez et al., 2016).

As the reasons part of the questionnaire was almost identical to the original one, we used CFA and ESEM in the total sample to test whether a three-factor dimension (instrumental, reactive, and defensive) accounted for the reasons reported by the adolescents for violent behaviors. The procedures for CFA and ESEM were similar to those described above. A post hoc analysis with semPower (Moshagen & Erdfelder, 2016) indicated that the power was high for all the models. For instance, the power for the bifactor ESEM model, with 93 degrees of freedom, was as follows: alpha = 0.05, RMSEA < 0.05, and *N* = 630 was > 0.999. Similarly, the power for the reasons model, with 403 degrees of freedom was as follows: alpha = 0.05, RMSEA < 0.05, and *N* = 1,244 was > 0.999.

## Results

### Violent Behaviors Scales

First, separate EFAs were conducted with the items of behaviors toward the mother and behaviors toward the father. In both cases, the results suggested a one-factor solution. For mothers, |λ| ranged between 0.61 and 0.87 (*M* = 0.76), and the fit indices were excellent (χ^2^ [27, *n* = 614] = 58, RMSEA = 0.043 [0.028–0.059], CFI = 0.982, TLI = 0.976). For fathers, |λ| ranged between 0.71 and 0.91 (*M* = 0.77), and the fit indices were adequate (χ^2^ [27, *n* = 614] = 78, RMSEA = 0.056 [0.04–1.070], CFI = 0.970, TLI = 0.959). The alpha ordinal coefficients were 0.93 for both CMV and CFV. Although a two-factor solution increased the fit indices for items focused on both mothers and fathers (χ2 [19, *n* = 614] = 32, RMSEA = 0.033 [0.010–0.053], CFI = 0.992, TLI = 0.986 and χ2 [19, *n* = 614] = 26, RMSEA = 0.025 [0.00–0.046], CFI = 0.996, TLI = 0.992, respectively), the solutions were not satisfactory. In both cases, items 1, 2, and 6 loaded on a second factor, but two of these items cross-loaded on the other factor as well. Therefore, these preliminary analyses supported the existence of a unidimensional structure for CMV and for CFV. These results were taken as a starting point for the subsequent analyses, which were carried out with all the items in subsample 2, including the behaviors toward the mother and father.

#### Nonhierarchical CFA and ESEM Models

Next, a two-factor correlated CFA model (CMV and CFV factors) and a two-factor ESEM model were calculated. The CFA model achieved excellent fit indices (χ^2^ [125, *n* = 630] = 243, RMSEA = 0.039 [0.032–0.046], CFI = 0.967, TLI = 0.960). The factor loadings were adequate for the CMV factor (|λ| range: 0.61 to 0.94, M = 0.77) and for the CFV factor (|λ| range: 0.45 to 0.81, M = 0.66). Correlation between factors was 0.74. The fit indices for the ESEM model were similar (χ^2^ [109, *n* = 630] = 214, RMSEA = 0.039 [0.031–0.047], CFI = 0.971, TLI = 0.960) and did not represent an improvement in fit indices (∆RMSEA and ∆TLI = 0, ∆CFI = 0.004). An examination of the factor loadings indicated that all but one (item 2) belonging to the CMV were significant and adequate (|λ| range: 0.17 to 0.99, M = 0.61). Several items focused on the father loaded significantly on the CMV factor. Overall, the CFV factor loadings were poor (|λ| range: − 0.05 to 0.78, M = 0.27). Five items focused on the father did not load significantly on the CFV factor, and several items focused on the mother loaded significantly on the CFV factor. In the ESEM model, the correlation between factors was reduced to 0.37. These results suggest that when items assessing CMV and CFV are integrated in the same model, there is a high overlap between the two. In the next step, using bifactor models we explored whether a general CPV factor could explain the data. Table 1 displays the factor loadings of all the measurement models.

**Table 1:** Factor loadings for the CFA, ESEM, bifactor CFA and bifactor ESEM Models

	ESEM			CFA		Bifactor ESEM				Bifactor CFA		
	CMVλ	CFVλ	Uniqueness	Specific λ	Uniqueness	CMVλ	CFVλ	General λ	Uniqueness	Specific λ	General λ	Uniqueness
CMV												
Mother 1	0.44^**^	0.43^**^	0.48	0.74^**^	0.45	0.59^**^	0.13	0.46^**^	0.42	0.51^**^	0.55^**^	0.44
Mother 2	**0.17**	0.77^**^	0.28	0.71^**^	0.49	0.75^**^	0.41^**^	0.26^*^	0.19	0.41^**^	0.57^**^	0.51
Mother 3	0.76^**^	0.25^*^	0.33	0.90^**^	0.34	0.57^**^	− 0.03	0.72^**^	0.30	0.61^**^	0.56^**^	0.31
Mother 4	0.74^**^	0.17	0.24	0.81^**^	0.30	0.50^**^	− 0.05	0.67^**^	0.21	0.61^**^	0.60^**^	0.29
Mother 5	0.90^**^	− 0.09	0.50	0.83^**^	0.49	0.30^*^	− 0.21^*^	0.81^**^	0.44	0.59^**^	0.55^**^	0.49
Mother 6	0.36^**^	0.49^**^	0.23	0.72^**^	0.19	0.60^**^	0.20^*^	0.40^**^	0.16	0.46^**^	0.66^**^	0.19
Mother 7	0.99^**^	− 0.05	0.01	0.94^**^	0.12	0.40^**^	− 0.18	0.89^**^	0.01	0.71^**^	0.65^**^	0.08
Mother 8	0.54^**^	0.16	0.62	0.61^**^	0.63	0.21^*^	0.11	0.58^**^	0.61	0.29^**^	0.53^**^	0.64
Mother 9	0.57^**^	0.15	0.59	0.64^**^	0.59	0.22^*^	0.11	0.61^**^	0.57	0.28^**^	0.57^**^	0.60
CFV												
Father 1	0.16	0.52^**^	0.65	0.60^**^	0.65	0.19^*^	0.50^**^	0.32^**^	0.62	0.37^**^	0.54^**^	0.57
Father 2	0.09	0.78^**^	0.34	0.71^**^	0.50	0.29^**^	0.69^**^	0.36^**^	0.31	0.44^**^	0.66^**^	0.36
Father 3	0.43^**^	0.37^*^	0.56	0.72^**^	0.47	0.04	0.48^**^	0.57^**^	0.52	**0.08**	0.72^**^	0.47
Father 4	0.60^**^	**0.15**	0.49	0.73^**^	0.48	0.08	0.21^*^	0.66^**^	0.43	**− 0.12**	0.70^**^	0.39
Father 5	0.73^**^	**− 0.05**	0.55	0.72^**^	0.58	− 0.07	**0.11**	0.75^**^	0.51	− 0.34^*^	0.61^**^	0.49
Father 6	0.15	0.60^**^	0.56	0.65^**^	0.48	0.23^**^	0.56^**^	0.36^**^	0.45	0.37^**^	0.72^**^	0.48
Father 7	0.90^**^	**− 0.14**	0.27	0.81^**^	0.34	0.18	**0.04**	0.92^**^	0.12	0.55^**^	0.83^**^	0.01
Father 8	0.48^**^	**0.13**	0.70	0.59^**^	0.66	0.03	0.25^*^	0.58^**^	0.61	**0.01**	0.59^**^	0.65
Father 9	0.38^*^	**0.10**	0.82	0.45^**^	0.80	− 0.20^*^	0.30^*^	0.48^**^	0.64	**0.03**	0.45^**^	0.80

Note. *p < .05, **p < .001; CPV = Child-to-Parent violence; CFV = Child-to-Father violence; CMV = Child-to-Mother violence

#### Bifactor Models

The bifactor CFA model obtained excellent fit indices (χ2 [108, *n* = 630] = 209, RMSEA = 0.039 [0.031–0.046], CFI = 0.972, TLI = 0.961). The factor loadings support the existence of a strong general factor of CPV (|λ| range: 0.45 and 0.83, *M* = 0.61). The specific CMV factor loadings were all significant (|λ| range: 0.28 to 0.71, *M* = 0.50), while those for the specific CFV factors were poor (|λ| range: − 0.34 to 0.55, M = 0.15). Four of the specific CFV factor loadings were not significant (3, 4, 8, 9) and two were negative. Thus, these results suggest that the general CPV factor accounts for the variance of several CFV items.

The fit indices for the bifactor ESEM model were excellent (χ^2^ [93, *n* = 630] = 114, RMSEA = 0.019 [0.00–0.030], CFI = 0.994, TLI = 0.990). This model increased fit indices compared to the ESEM model (∆RMSEA = 0.020, ∆TLI = 0.029, ∆CFI = 0.022). All factor loadings on the general CPV factor were statistically significant (|λ| range: 0.26 to 0.92, *M* = 0.58). In addition, all factor loadings on the CMV factor were statistically significant (|λ| range: 0.21 to 0.75, *M* = 0.46). Meanwhile, all but two of the factor loadings on the CFV (items 5 and 7) were statistically significant (|λ| range: 0.04 to 0.69, *M* = 0.35).

Overall omega (ω) and omega subscale (ωs) values were high, which indicates that the proportion of the variance of both the CPV and the specific CMV and CFV factors explained by all sources of common variance was high. ωh was adequate (> 0.70) in both bifactor models, which indicates that the general CPV factor explains a high proportion of the total scores. Conversely, ωhs values were low, indicating that the reliability of the subscales (CMV and CFV) is poor after controlling for the variance related to the general factor. In the bifactor ESEM model, H values were higher than 0.70 for the general factor and the CMV and close to 0.70 in the case of CFV, which suggests replicability across samples (Rodriguez et al., 2016). In the bifactor CFA model, this criterion was not satisfied for CFV. The PUC and ECV values were under the cutoff of 0.70. The omega coefficients are included as Supplementary Material (10.17605/OSF.IO/CDJUW).

### Reasons for the Aggressions

As the items about reasons for the aggressions were very similar to the original version of the CPAQ, CFA and ESEM models were used with the full sample to test the existence of three factors: instrumental reasons (items 1 and 2), reactive reasons (items 3, 6 and 7), and defensive reasons (items 4 and 5). The fit indices were similar (∆RMSEA = 0.013; ∆CFI = 0.006; ∆TLI = 0.004). The factor loadings are shown in Table 2. In general, the factor loadings are strong in both the CFA and ESEM models on their corresponding factors (|λ| range: 0.62 to 0.98, *M* = 0.78 and |λ| range: 0.46 to 0.96, *M* = 0.74, for the CFA and ESEM model, respectively). In the ESEM model, the cross-loadings tend to be non-significant and very small. The exceptions are reason 4 (to defend other people, which tends to load on reactiveness) and reason 7 (because my parents wanted me to do something that bothered me). The correlations between factors obtained with both models were very similar: between 0.39 and 0.75 for the CFA model and between 0.32 and 0.59 for the ESEM model. Thus, there was no reason to select the ESEM model, and the CFA was preferred because it provides a more parsimonious explanation of the reasons for the aggressions. The ordinal alpha coefficients were 0.87, 0.87, and 0.90, respectively, for instrumental, reactive, and defensive reasons.

**Table 2:** Factor loadings for the reason scales

	ESEM				CFA model	
	1	2	3	Uniqueness		Uniqueness
1. Instrumental						
Reason 1 mother	**0.94** ^**^	0.11^*^	− 0.07	0.11	0.98^**^	0.04
Reason 1 father	**0.93** ^**^	0.15^*^	− 0.13	0.15	0.97^**^	0.07
Reason 2 mother	**0.65** ^**^	− 0.14^*^	0.18	0.50	0.64^**^	0.59
Reason 2 father	**0.66** ^**^	− 0.15^*^	0.15	0.51	0.62^**^	0.62
2. Reactive violence						
Reason 3 mother	− 0.08	**0.78** ^**^	0.04	0.39	0.76^**^	0.42
Reason 3 father	− 0.08	**0.82** ^**^	0.03	0.34	0.75^**^	0.44
Reason 6 mother	− 0.03	**0.78** ^**^	0.06	0.35	0.77^**^	0.40
Reason 6 father	0.04	**0.71** ^**^	0.06	0.39	0.79^**^	0.37
Reason 7 mother	0.23^**^	**0.46** ^**^	0.09	0.59	0.68^**^	0.53
Reason 7 father	0.24^**^	**0.51** ^**^	0.04	0.58	0.64^**^	0.58
3. Defensive reason						
Reason 4 mother	− 0.02	0.31^**^	**0.59** ^**^	0.34	0.91^**^	0.18
Reason 4 father	− 0.02	0.24^**^	**0.65** ^**^	0.35	0.88^**^	0.22
Reason 5 mother	− 0.01	− 0.13^*^	**0.96** ^**^	0.17	0.76^**^	0.42
Reason 5 father	0.01	− 0.09	**0.94** ^**^	0.21	0.76^**^	0.42

Note. * *p* < .05, ** *p* < .001. The bolded coefficients represent specific coefficients for each factor

Finally, a structural equation model was estimated to examine the associations between the latent variables. The fit indices were adequate (χ2 [403, *N* = 1244] = 602, RMSEA = 0.028 [0.023–0.033], CFI = 0.976, TLI = 0.970). The general CPV factor was significantly associated with instrumental reasons (0.52, *p* < .001), reactive reasons (0.56, *p* < .001), and defensive reasons (0.58, *p* < .001). Regarding the associations of CFV and CMV with specific factors, the only statistically significant associations were between reactive reasons and CMV (0.38, *p* < .001) and CFV (0.20, *p* < .002), which means that the associations between instrumental and defensive reasons and the specific CMV and CFV factors are explained by their association with the general CPV.

### Frequency of CPV During the Pandemic and Differences According to the Sex of Adolescents and Parents

Table 3 shows the percentage of adolescents who indicated at least one behavior from each item of the questionnaire. For sex differences, we did not include adolescents who selected a binary category for sex because they were only 11 and the percentage could not be representative. For most of the items, there were no significant differences according to sex. The exceptions were insulting, which was more frequent in girls than in boys, and breaking an object in an argument, which was more frequent in boys than in girls.

**Table 3:** Percentages of participants who endorsed at least one behavior by item

	Mother						Father			
	Total	Boys	Girls	χ^2^	*p*	Total	Boys	Girls	*χ*^*2*^	*p*
1. You’ve made fun of him/her	15.8	15	16.6	0.57	0.482	17.6	16.1	19.1	1.91	0.096
2. You’ve said something humiliating or disqualifying to him/her	20.5	17.9	23.1	5.05	0.028	23.4	21.8	25.4	2.15	0.156
3. You’ve threatened to hit him/her, even though you didn’t actually do it	5	5.5	4.4	0.86	0.214	4.6	5.1	4.1	0.63	0.494
4. You’ve pushed or hit him/her in a fight	5.8	5.4	6.2	0.36	0.318	5.9	5.6	6.1	0.16	0.715
5. You’ve hit him/her with something that could hurt	2.4	2.1	2.6	0.32	0.354	3.9	4.3	3.5	0.52	0.552
6. You have insulted him/her	34.8	28.8	40.8	19.45	< 0.001	35	30.5	39.4	10.48	0.001
7. You’ve kicked or punched him/her	2.3	2.4	2.1	0.15	0.849	3.8	3.9	3.6	0.07	0.881
8. You have broken an object of the home in an argument with him/her	10.5	12.6	8.5	5.51	0.020	10.6	13.6	7.6	11.47	< 0.001
9. You’ve made him/her feel scared in an argument	11.6	11.7	11.4	0.04	0.859	8	8.5	7.5	0.49	0.526
Reasons										
1. To get permission for something (e.g., time to get home, to be able to use the mobile, …)	41.9	41.8	42.1	0.01	0.945	37.6	38.5	36.9	0.25	0.619
2. Because I needed money	24.2	25.4	23.1	0.61	0.470	21.6	22.3	21	0.20	0.675
3. Because I was very angry	51.8	46.3	56.5	8.86	0.003	51.1	44.6	56.8	12.60	< 0.001
4. To defend myself	25.4	22.1	28.3	4.37	0.040	26	21.3	30.2	8.52	0.004
5. To defend another person	24.9	24.9	24.9	0	1	25.8	24.1	27.4	1.16	0.304
6. Because I lost control	30.3	30.9	29.8	0.14	0.709	29.4	27.5	28.4	0.37	0.591
7. Because they were bugging me asking me to do something.	40.5	39.7	41.1	0.17	0.726	35	33.8	36.2	0.54	0.469

Table 4 shows the percentages of adolescents who engaged in at least one behavior and those who repeatedly engaged in some form of violence. For reiterate violence, the percentage of adolescents who reported any violent behavior at least six times in the last year was considered. There were no statistically significant differences based on sex in reiterative behaviors. However, compared to boys, in girls the percentage of adolescents who engaged in at least one behavior was slightly higher.

**Table 4:** Prevalence rates of violence against mothers and fathers

	Total *N* = 1,244	Boys *N* = 618	Girls *N* = 615	χ^2^	*p*
Against mother (at least one behavior)	49.9	45.8	53.8	7.94	0.005
Against father (at least one behavior)	51.8	48.8	54.7	4.28	0.039
Total against both mother and father (at least one behavior)	60.3	56.8	63.7	6.21	0.014
Against mother (reiterative)	10.6	9.5	11.5	1.30	0.267
Against father (reiterative)	11.9	11.4	12.5	0.35	0.597
Total against both mother and father (reiterative)	16.5	14.9	18	0.15	0.145

Comparison of the frequency of CMV with CFV using *t-tests* for paired samples indicated that there were no statistically significant differences (*p* = .281). There were also no significant differences in the percentages of CMV and CFV for both the total mean score (*z* = 1.56, *p* = .120) and reiterative behaviors (*z* = 1.40, *p* = .162).

## Discussion

The main objective of this study was to develop an updated version of the CPAQ, a questionnaire that is widely used to assess CPV behaviors (Arias-Rivera et al., 2020). The results provide data on the dimensionality and reliability of the revised CPAQ in Spanish adolescents. In addition, the study helps us to understand the magnitude of CPV in the context of the COVID-19 pandemic.

Like the original version of the CPAQ, the new version includes two sections: one on violent behaviors and the other on reasons for these behaviors. In terms of behaviors, the new version is better aligned with the definition of CPV based on the consensus of a committee of expert CPV clinicians and researchers (Pereira et al., 2017) and the stories of families involved in CPV (Calvete et al., 2015d), as it eliminated items that represent rebellious and rule-breaking behaviors that are relatively common in adolescence, such as yelling at a parent when angry or doing something to intentionally annoy the parent. Due to the elimination of these items and the inclusion of new ones (e.g., “made him/her feel scared in an argument”), the new version can be considered a stricter measure of violence against parents.

Regarding the dimensionality of violent behaviors, the results of numerous factor analyses supported a bifactor ESEM model in which a general factor of violence against parents would explain aggression against both mothers and fathers. The measurement model explained a significant percentage of the variance of both total CPV and CMV and CFV scores. According to this model, the specific dimensions of CMV and CFV explain little beyond what is explained by the general factor of CPV. This measurement model supports the use of a measure obtained from the 18 parallel items of the questionnaire. The general dimension exhibited an excellent reliability index. However, the results obtained using EFA and CFA show that the use of the separate CMV and CFV scales is also possible. Indeed, these scales obtained excellent ordinal alpha coefficients. The use of these specific scales may be necessary in studies where their role is to be compared or in single-parent family contexts.

The most frequent aggressive behaviors toward parents were insulting, humiliating or disqualifying, and ridiculing (i.e., psychological violence behaviors). These were followed in frequency by intimidating behaviors, such as making the parent feel scared in an argument and breaking an object in the home during an argument with the parent. Finally, physical aggressions were the least frequent (e.g., hit the parent with something that could hurt or kick or punch him/her). These results are consistent with the findings of previous research (Simmons et al., 2018).

The questionnaire also assesses the reasons for aggression. Consistent with the original version (Calvete & Orue, 2016), factor analyses confirmed the structure consisting of three types of reasons: instrumental, reactive, and defensive. Instrumental reasons involve the use of aggression by the adolescent to obtain a benefit and have been identified as antecedents of CPV (Calvete et al., 2013b) and associated with the culture of consumerism (Calvete & Orue, 2016). Reactive reasons include anger, which has been proposed as a proximal trigger of CPV (Calvete et al., 2015a). Finally, defensive reasons include both self-defense and defense of another family member. Defensive reasons are consistent with the association between CPV and previous exposure to family violence (Calvete et al., 2015c). In addition, reactive reasons were also associated with CMV- and VFC-specific factors. This suggests that aggressions against mothers and fathers shared the elements of instrumentality and defensiveness, as the association between these reasons and specific behaviors against mothers and fathers was explained by their association with the general factor of CPV. That is, reasons such as obtaining money or privilege and defensiveness simultaneously characterized CMV and CFV. In contrast, the analyses showed that there was some specificity in relation to reactive reasons, which were somewhat more strongly associated with aggression toward mothers. The reasons subscales showed good internal consistency. The procedure for assessing the reasons is greatly simplified in the new version, saving time and effort.

Regarding the sex of the adolescent, there were no differences in the prevalence of repeated violence toward either parent based on sex. However, there were a few item-level differences. Specifically, higher rates of humiliating and disqualifying both parents and insulting the mother were found in girls, and higher rates of breaking objects during arguments were found in boys. The higher frequency of girls reporting the aforementioned psychological aggressions is consistent with the findings of previous studies indicating a higher prevalence of psychological aggressions by girls, particularly against their mother (Calvete et al., 2013a).

Regarding the sex of the parents, there were no differences in aggressions against mothers and fathers. This result contrasts markedly with those obtained in previous research, which point to a higher frequency of aggressions against mothers than against fathers (Simmons et al., 2018). In fact, the higher frequency of aggression toward mothers in previous studies motivated the creation of questionnaires for the assessment of CPV exclusively against mothers (Edenborough et al., 2011). These results should be interpreted in light of the COVID-19 pandemic. Data were collected between October 2020 and April 2021. Since adolescents reported the frequency of behaviors during the previous year, this implies that the assessment period included the first three waves of the pandemic. Specifically, the first wave involved strict lockdown of the entire population from March to May 2020. Although the second and third waves did not involve total confinement in Spain, the state of alarm was maintained, and mobility was severely restricted. Thus, for many weeks, citizens had to return to their homes earlier than usual, and this, together with the increased frequency of teleworking, increased the number of hours of cohabitation of family members at home. This situation led fathers to spent more time at home with their children and, therefore, to greater involvement in parenting and discipline. This may have brought fathers into the zone of conflict with their children in which CPV episodes are usually triggered, thus contributing to an increase in aggression against fathers. This may explain the relatively high rates of repeated violence against parents (16.5% of adolescents) compared to pre-pandemic studies. For example, in another sample of Spanish adolescents, Calvete et al. (2017) found rates of 9.4% for reiterative psychological violence against mothers and 8.5% against fathers and rates of 6.1% for reiterative physical violence against mothers and 5.1% against fathers. Therefore, the scenario created by the pandemic offers data that may contribute to a better understanding of CPV and suggests that the differences previously found regarding the sex of the parent could mainly be due to the greater presence of mothers in parenting.

The COVID-19 pandemic situation could also be on the basis of relatively high rates of repeated violence against parents (16.5% of adolescents). Several experts have highlighted factors that may have contributed to increased family violence during the COVID-19 pandemic, such as reduced space for family activities and a narrow buffer zone for conflicts (Zhang, 2022), changes in structures and routines, and a lack of access to formal and informal support (Condry et al., 2020). However, other explanations for the increase in aggressions against parents cannot be ruled out. According to the available data, intimate partner violence against mothers (Arenas-Arroyo et al., 2021) and family violence in general (Drotning et al., 2022) have also increased during the COVID-19 pandemic. Therefore, the greater presence of fathers in the homes could imply an increase in child maltreatment and intimate partner violence against mothers, and both could act as antecedents to CPV (Calvete et al., 2015c; Ibabe et al., 2020), In fact, between 24 and 30% of adolescents indicated self-defense or defense of another person as a motive for the aggressions. In any case, the data from this study reinforce the key impact of presence in the home and involvement in parenting on who becomes a target of assaults.

### Limitations and Strengths

This study has some limitations that should be considered when evaluating its conclusions. First, only the adolescents’ self-report version of the CPAQ was used. Future studies should use the new version adapted to parents in order to consider the perspectives of all persons involved. For example, in a study comparing adolescent reports with those of their parents, it was found that the prevalence rates obtained through parental reports were generally lower than those obtained when asking their children (Calvete et al., 2017). Therefore, it would be interesting to consider the point of view of both children and parents. A second limitation concerns the cross-sectional nature of the study. It would be advisable to include test–retest measures to determine the temporal reliability of the CPAQ. Longitudinal studies are also important to study the trajectories of CPV (e.g., Calvete et al., 2020). Third, the study was conducted in a sample of community adolescents. It would be advisable for future studies to use samples from other populations, such as judicial or clinical samples. The study’s strengths include the sample size and the provision of data on CPV during the COVID-19 pandemic.

In summary, this study presents a revised and improved version of the CPAQ for assessing violence perpetrated by adolescents and children against their parents. This questionnaire assesses a general dimension of CPV and has excellent psychometric properties. Therefore, we believe that this questionnaire could be used in both research and clinical settings. Moreover, the data indicate a high incidence of aggressions against parents during the COVID-19 pandemic, with similar rates for fathers and mothers.

## References

[CR1] Arenas-Arroyo E, Fernandez-Kranz D, Nollenberger N (2021). Intimate partner violence under forced cohabitation and economic stress: Evidence from the COVID-19 pandemic. Journal of Public Economics.

[CR2] Arias-Rivera S, Hidalgo V, Lorence B (2020). A scoping study on measures of child-to-parent violence. Aggression and Violent Behavior.

[CR3] Armstrong GS, Cain CM, Wylie LE, Muftić LR, Bouffard LA (2018). Risk factor profile of youth incarcerated for child to parent violence: A nationally representative sample. Journal of Criminal Justice.

[CR4] Asparouhov T, Muthén B (2009). Exploratory structural equation modeling. Structural Equation Modeling: A Multidisciplinary Journal.

[CR5] Brule N, Eckstein JJ (2016). “Am I really a bad parent?”: Adolescent-to-Parent Abuse (AtPA) identitiy and the Stigma Management Communication (SMC) model. Journal of Family Communication.

[CR6] Calvete, E. (2019). Evaluación de la violencia filio-parental. In E.Calvete & R.Pereira (Eds.). *La violencia Filio-parental: Análisis, evaluación a intervención*. Alianza.

[CR7] Calvete, E., Gámez-Guadix, M., Orue, I., Gonzalez-Diez, Z., Lopez de Arroyabe, E., Sampedro, R., … Borrajo, E. (2013a). Brief report: The adolescent child-to-parent aggression questionnaire: An examination of aggressions against parents in Spanish adolescents. *Journal of Adolescence,**36*, 1077–1081. 10.1016/j.adolescence.2013.08.017 10.1016/j.adolescence.2013.08.017 24215954

[CR8] Calvete E, Orue I, Gámez-Guadix M (2013). Child-to-parent violence: emotional and behavioral predictors. Journal of Interpersonal Violence.

[CR9] Calvete E, Orue I (2016). Child-to-parent violence: Frequency and reasons for the aggressions against fathers and mothers. Behavioral Psychology.

[CR10] Calvete E, Orue I, Fernández-González L, Chang R, Little TD (2020). Longitudinal trajectories of child-to-parent violence through adolescence. Journal of Family Violence.

[CR11] Calvete E, Gámez-Guadix M, García-Salvador S (2015). Social information processing in child-to-parent aggression: Bidirectional associations in a 1-year prospective study. Journal of Child and Family Studies.

[CR12] Calvete E, Orue I, Gámez-Guadix M (2015). Reciprocal longitudinal associations between substance use and child-to-parent violence in adolescents. Journal of Adolescence.

[CR13] Calvete E, Orue I, Gámez-Guadix M, Bushman BJ (2015). Predictors of child-to-parent aggression: A 3-year longitudinal study. Developmental Psychology.

[CR14] Calvete E, Orue I, Gámez-Guadix M, del Hoyo-Bilbao J, de Arroyabe EL (2015). Child-to-parent violence: An exploratory study of the roles of family violence and parental discipline through the stories told by Spanish children and their parents. Violence and Victims.

[CR15] Calvete E, Orue I, González-Cabrera J (2017). Violencia filio parental: comparando lo que informan los adolescentes y sus progenitores. Revista de Psicología Clínica con Niños y Adolescentes.

[CR16] Cano-Lozano M, Navas-Martínez MJ, Contreras L (2021). Child-to-parent violence during confinement due to COVID-19: Relationship with other forms of family violence and psychosocial stressors in Spanish youth. Sustainability.

[CR17] Chen FF (2007). Sensitivity of goodness of fit indexes to lack of measurement invariance. Structural Equation Modeling: A Multidisciplinary Journal.

[CR18] Cheung GW, Rensvold RB (2002). Evaluating goodness-of-fit indexes for testing measurement invariance. Structural Equation Modeling.

[CR19] Condry R, Miles C (2014). Adolescent to parent violence: Framing and mapping a hidden problem. Criminology & Criminal Justice.

[CR20] Condry, R., Miles, C., Brunton-Douglas, T., & Oladapo, A. (2020). *Experiences of child and adolescent to parent violence in the Covid-19 pandemic*. University of Oxford. https://www.law.ox.ac.uk/sites/files/oxlaw/final_report_capv_in_covid-19_aug20.pdf

[CR21] Del Hoyo-Bilbao J, Gámez-Guadix M, Orue I, Calvete E (2018). Psychometric properties of the Child-to-Parent Aggression Questionnaire in a clinical sample of adolescents who abuse their parents: Prevalence and gender differences. Violence and Victims.

[CR22] Del Hoyo-Bilbao J, Orue I, Gámez-Guadix M, Calvete E (2020). Multivariate models of child-to-mother violence and child-to-father violence among adolescents. The European Journal of Psychology Applied to Legal Context.

[CR23] Drotning KJ, Doan L, Sayer LC, Fish JN, Rinderknecht RG (2022). Not all homes are safe: Family violence following the onset of the covid-19 pandemic. Journal of Family Violence.

[CR24] Edenborough M, Wilkes LM, Jackson D, Mannix J (2011). Development and validation of the Child-to-Mother Violence Scale. Nurse Research.

[CR25] Flurry LA, Swimberghe K, Allen J (2021). Exposing the moderating impact of parent-child value congruence on the relationship between adolescents’ materialism and subjective well-being. Journal of Business Research.

[CR26] Fundación, A. (2021). *La violencia filio-parental en España (datos 2020)*. https://fundacionamigo.org/wp-content/uploads/2021/09/vfp2021.pdf

[CR27] Hammer, J. H., & Toland, M. D. (2016). *Bifactor analysis in Mplus*. http://sites.education.uky.edu/apslab/upcoming-events/

[CR28] Ibabe I (2019). Adolescent-to-parent violence and family environment: The perceptions of same reality?. International Journal of Environmental Research and Public Health.

[CR29] Ibabe, I. (2020). A systematic review of youth-to-parent aggression: conceptualization, typologies, and instruments. *Frontiers in Psychology*, 3240. 10.3389/fpsyg.2020.577757. 10.3389/fpsyg.2020.577757 PMC7734055 33329226

[CR30] Ibabe I, Arnoso A, Elgorriaga E (2014). Behavioral problems and depressive symptomatology as predictors of child-to-parent violence. The European Journal of Psychology Applied to Legal Context.

[CR31] Ibabe I, Arnoso A, Elgorriaga E (2020). Child-to-parent violence as an intervening variable in the relationship between inter-parental violence exposure and dating violence. International Journal of Environmental Research and Public Health.

[CR32] Ibabe I, Bentler P (2016). The contribution of family relationships to child-to-parent violence. Journal of Family Violence.

[CR33] Ibabe I, Jaureguizar J (2010). Child-to-parent violence: Profile of abusive adolescents and their families. Journal of Criminal Justice.

[CR34] Little TD (2013). Longitudinal structural equation modeling.

[CR35] Margolin G, Baucom BR (2014). Adolescents’ aggression to parents: Longitudinal links with parents’ physical aggression. Journal of Adolescent Health.

[CR36] Markon KE (2019). Bifactor and hierarchical models: Specification, inference, and interpretation. Annual Review of Clinical Psychology.

[CR37] Morin AJ, Arens AK, Marsh HW (2016). A bifactor exploratory structural equation modeling framework for the identification of distinct sources of construct-relevant psychometric multidimensionality. Structural Equation Modeling: A Multidisciplinary Journal.

[CR38] Moshagen M, Erdfelder E (2016). A new strategy for testing structural equation models. Structural Equation Modeling.

[CR39] Muthén LK, Muthén BO (2021). Mplus user’s guide.

[CR40] O’Hara KL, Duchschere JE, Beck CJA, Lawrence E (2017). Adolescent-to-parent violence: Translating research into effective practice. Adolescent Research Review.

[CR41] Pagani L, Tremblay R, Nagin D, Zoccolillo M, Vitaro F, McDuff P (2009). Risk factor models for adolescent verbal and physical aggression toward fathers. Journal of Family Violence.

[CR42] Passini S (2013). A binge-consuming culture: The effect of consumerism on social interactions in western societies. Culture & Psychology.

[CR43] Pereira R, Loinaz I, Del Hoyo-Bilbao J, Arrospide J, Bertino L, Calvo A, Gutiérrez MM (2017). Proposal for a definition of filio-parental violence: Consensus of the Spanish society for the study of filio-parental violence. Psychologist Papers.

[CR44] Reise SP, Scheines R, Widaman KF, Haviland MG (2013). Multidimensionality and structural coefficient bias in structural equation modeling: A bifactor perspective. Educational and Psychological Measurement.

[CR45] Rodriguez A, Reise SP, Haviland MG (2016). Evaluating bifactor models: Calculating and interpreting statistical indices. Psychological Methods.

[CR46] Simmons M, McEwan TE, Purcell R, Ogloff JR (2018). Sixty years of child-to-parent abuse research: What we know and where to go. Aggression and Violent Behavior.

[CR47] Straus, M. A., & Fauchier, A. (2008). *The international parenting study*. http://pubpages.unh.edu/~mas2/IPS.htm

[CR48] Straus MA, Hamby SL, Boney-McCoy S, Sugarman DB (1996). The revised conflict tactics scales (CTS2): Development and preliminary psychometric data. Journal of Family Issues.

[CR49] Tabachnick, B. G., Fidell, L. S., & Ullman, J. B. (2007). *Using multivariate statistics* (5th Ed.). Pearson.

[CR50] Ulman A, Straus MA (2003). Violence by children against mothers in relation to violence between parents and corporal punishment by parents. Journal of Comparative Family Studies.

[CR51] Usher K, Bradbury Jones C, Bhullar N, Durkin DJ, Gyamfi N, Fatema SR, Jackson D (2021). COVID-19 and family violence: Is this a perfect storm?. International Journal of Mental Health Nursing.

[CR52] Watkins MW (2013). Omega. [Computer software].

[CR53] Williams M, Tuffin K, Niland P (2017). “It’s like he just goes off, BOOM!”: Mothers and grandmothers make sense of child-to-parent violence. Child and Family Social Work.

[CR54] Yeager DS, Dahl RE, Dweck CS (2018). Why interventions to influence adolescent behavior often fail but could succeed. Perspectives on Psychological Science.

[CR55] Zhang H (2022). The influence of the ongoing COVID-19 pandemic on family violence in China. Journal of Family Violence.

